# Tunable quantum dots in monolithic Fabry-Perot microcavities for high-performance single-photon sources

**DOI:** 10.1038/s41377-024-01384-7

**Published:** 2024-01-30

**Authors:** Jiawei Yang, Yan Chen, Zhixuan Rao, Ziyang Zheng, Changkun Song, Yujie Chen, Kaili Xiong, Pingxing Chen, Chaofan Zhang, Wei Wu, Ying Yu, Siyuan Yu

**Affiliations:** 1 State Key Laboratory of Optoelectronic Materials and Technologies, School of Electronics and Information Technology, Sun Yat-Sen University, Guangzhou, 510006 China; 2 Institute for Quantum Science and Technology, College of Science, National University of Defense Technology, Changsha, 410073 China; 3 College of Advanced Interdisciplinary Studies, National University of Defense Technology, Changsha, 410073 China; 4 Hunan Key Laboratory of Quantum Information Mechanism and Technology, National University of Defense Technology, Changsha, 410073 Hunan China; 5 Hefei National Laboratory, Hefei, 230088 China

**Keywords:** Quantum dots, Photonic devices, Quantum optics

## Abstract

Cavity-enhanced single quantum dots (QDs) are the main approach towards ultra-high-performance solid-state quantum light sources for scalable photonic quantum technologies. Nevertheless, harnessing the Purcell effect requires precise spectral and spatial alignment of the QDs’ emission with the cavity mode, which is challenging for most cavities. Here we have successfully integrated miniaturized Fabry-Perot microcavities with a piezoelectric actuator, and demonstrated a bright single-photon source derived from a deterministically coupled QD within this microcavity. Leveraging the cavity-membrane structures, we have achieved large spectral tunability via strain tuning. On resonance, a high Purcell factor of ~9 is attained. The source delivers single photons with simultaneous high extraction efficiency of 0.58, high purity of 0.956(2) and high indistinguishability of 0.922(4). Together with its compact footprint, our scheme facilitates the scalable integration of indistinguishable quantum light sources on-chip, therefore removing a major barrier to the development of solid-state quantum information platforms based on QDs.

## Abstract (web summary)

We demonstrate a high-performance single-photon source based on a monolithic FP microcavity, and the thin-film microcavity structure facilitates effective strain transduction.

## Graphical abstract

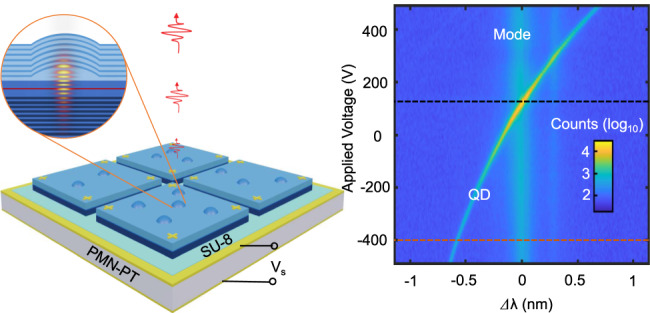

## Introduction

The development of quantum light sources that are capable of deterministically producing efficient and indistinguishable photonic states, is crucial for both exploring fundamental quantum physics and various applications, ranging from quantum communication^1^, photonic quantum computing^2^ and quantum metrology^3^. Among the myriad material platforms available^4–8^, semiconductor quantum dots (QDs) offer a promising way to create single-photons on demand^9^. In addition, the manipulation of spin states in confined electrons/holes^10^ or dark exciton^11^ within QDs enables the creation of multi-photon entanglement, paving the way for cluster photon states^12,13^. However, as a solid-state system, QDs in intrinsic bulk material face challenges such as low photon indistinguishability^14^ and low collection efficiency^15^, posing a significant bottleneck in their practical utilization.

A widely adopted approach to circumvent this problem is to embed QDs into photonic cavities^15–19^. Coupling QDs, both spectrally and spatially, with the cavity mode enhances and redirects the light. Both high extraction efficiency and high indistinguishability can be achieved simultaneously for well-designed cavities. Among these architectures, open cavities and micro-pillar photonic cavities hold great promise to generate indistinguishable photons efficiently^20,21^. In an open microcavity, spectral alignment is achieved by moving the separated mirror using nano-positioners^17^. However, this approach inevitably introduces a highly sophisticated system, particularly in terms of its sensitivity to mechanical vibrations. Furthermore, scaling up these systems remains challenging due to their large footprint size. While in the micro-pillar systems, spectral overlap is typically achieved through temperature tuning^9,22,23^ or the optical Stark effect^18,24^, both of which are not preferred as they can degrade the quality of the photon source. Strain tuning presents an attractive alternative, allowing the tailoring of various QD properties in a large range without observable degradation of photon coherence or brightness^25,26^. Despite its appeal, the implementation of strain tuning remains challenging, particularly in micro-pillars, due to their isolated structure and high aspect ratio.

Here we present a novel monolithic Fabry-Perot microcavity structure that combines deterministic fabrication, spectral-tunability and a high Purcell effect to efficiently generate single photons. We utilize a fabrication process to integrate a positioned QD in a Fabry-Perot microcavity with a piezoelectric actuator. A small mode volume is achieved via strong lateral mode confinement facilitated by a micrometer-scale parabolic lensed defect between two distributed Bragg reflectors (DBRs)^27^. The microcavity membrane structures allow compensation of the spectral mismatch between QD emissions and the cavity mode via a strain field^28–32^. This is achieved by integrating QDs onto a piezo actuator. The resulting compact and mechanically robust device makes our source less susceptible to external acoustic noise, compared to an open cavity consisting of two separate DBRs. The Purcell effect enables the generation of polarized single photons with simultaneous high extraction efficiency, high purity and photon indistinguishability. We anticipate that our unique capability to create well-ordered, tunable quantum light sources at the micrometer scale on a single substrate will significantly contribute to the advancement of scalable quantum information processing.

## Results

### Design concept

**Fig. 1:**
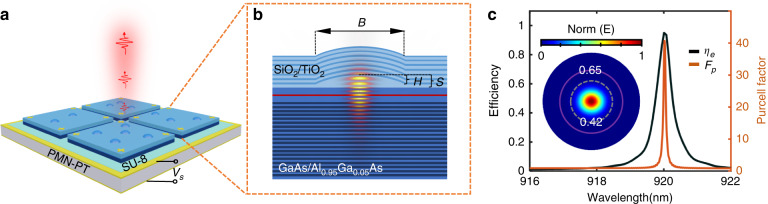
The design of strain tunable single photon source. **a** Sketch of tunable single photon source. Transferable Fabry-Perot microcavity integrated with PMN-PT (100) substrate by SU-8. **b** Cross-section of monolithic Fabry-Perot microcavity and electric field distribution of fundamental mode. The vertical confinement is from two mirrors: The top one is a dielectric SiO_2_/TiO_2_ distributed Bragg reflector (DBR), while the bottom one consists of GaAs/AlGaAs DBR. The lateral confinement is provided by the parabolic lensed-defect in the central spacer layer. Here, B is the base width of lensed defect, H is the height and S is the thickness of total SiO_2_ spacer. The InAs/GaAs QD is positioned at the field maximum within the GaAs microcavity. **c**. Three-dimensional simulation result of the microcavity. Extraction efficiency *η*_e_ of ~0.949 and Purcell factor *F*_p_ of ~40 are obtained in device with *B* = 4 μm, *H* = 350 nm, and *S* = 480 nm for 7-pairs top DBR. Inset is the far-field distribution showing near Gaussian profile. Dotted gray and solid purple circles represent NA = 0.42 and NA = 0.65, respectively

To realize bright, tunable quantum light sources with enhanced emission rates via the Purcell effect, we introduce a monolithic Fabry-Perot microcavity. This structure offers distinct advantages, including optimal utilization of the Purcell effect, a minimized footprint, and adeptness for integration. As schematically depicted in Fig. 1a, the configuration comprises a parabolic lensed defect within a Fabry-Perot DBR cavity, positioned atop a (100)-cut [Pb(Mg_1/3_Nb_2/3_)O_3_]_0.72_[PbTiO_3_]_0.28_ (PMN-PT) piezoelectric actuator. The microcavity membrane, with a flat morphology and a thickness of ~9 μm, facilitates efficient strain transfer. Within the microcavity, photons experience vertical confinement by top and bottom DBRs, while lateral confinement arises from the parabolic lensed defect in the central spacer layer, as illustrated in the spatial distribution of the electric field (*E*_r_) in Fig. 1b.

The concept of a Gaussian-shaped defect Fabry-Perot cavity was first introduced in 2013^27^. Recent efforts focused on implementing such a cavity within the GaAs material system for the utilization of QDs as single photon sources^33^. However, as a result of various growth rates along the unique crystal axis, the epitaxial layers of the top mirror become flattened after ~3 pairs-layer growth and the final cavities feature an elliptical shape. In our approach, we address this issue by employing dielectric layers as the top DBR. This allows precise engineering of the cavity resonance by adjusting both the shape of the defect and the cavity’s thickness (for more information, refer to Supplementary Information Section 1-I and Fig. S1). Moreover, the fundamental mode of the cavity exhibits a Gaussian-like far-field pattern (as seen in the inset of Fig. 1c), similar to micropillars, enabling efficient single photon extraction into free-space or single-mode fibers. For the QDs positioned in the center of the cavity, we can attain an extraction efficiency η_e_ as high as 94.9% with a Purcell factor of 40, using a 7-pair top DBR and a 46-pair bottom DBR, as shown in Fig. 1c (see details in Supplementary Information Section 1-I). With such a high factor, we can determine a near unity mode coupling efficiency $\beta$~0.975, calculated using the formula $\beta$=*F*_p_/(*F*_p_+1), where *F*_P_ represents the Purcell factor.

### Device fabrication and characterization

**Fig. 2:**
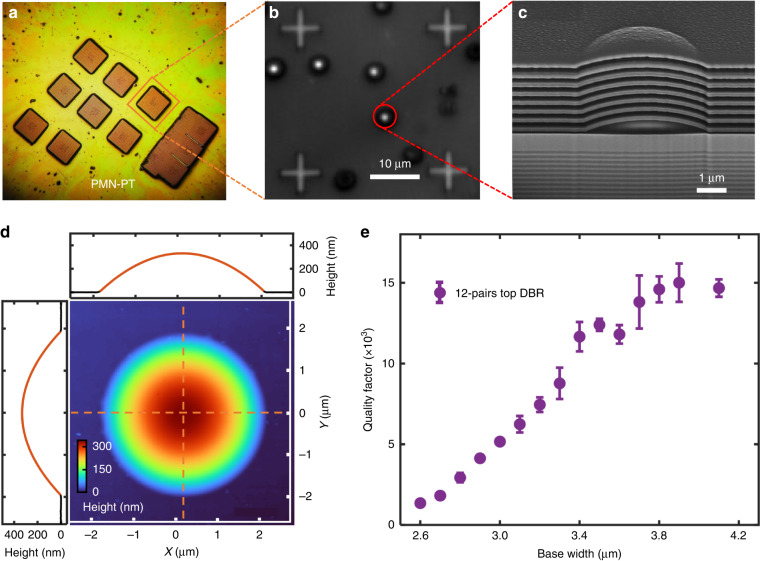
Integrated demo and cavity characterization. **a** Transferable films with cavities are glued to PMN-PT (100) substrate by SU-8 adhesive. Each square film is in size of ~280 μm × 280 μm. **b** Wide-field photoluminescence image of the fabricated Fabry-Perot microcavities with a single quantum dot in each center. The emissions from quantum dots (QDs) are excited by a high-power blue LED (445 nm), while the markers are illuminated by a white LED. The adjacent markers are separated by a distance of 30 μm. **c** Scanning microscope image of the cross-section of the cavity, which is milled by focus ion beam. The lens-shaped structure is maintained even after depositing an 8-pair dielectric DBR (~2 μm thick) on top. **d** Atomic force microscope analysis: The color map demonstrates a near rotationally symmetric lens-shaped defect. XZ (YZ) profile is depicted in the upper (left) panel (dotted black line) and fitted to a parabola, respectively. The derived dimensions of the defect include a base width of 3.8 (3.9) μm and a height of 335 nm. One standard deviation of ~0.8 (1.3) nm of the fitted lines is extracted, revealing the paraboloid nature of the defect. **e** Quality factor measurement: The quality factor (Q-factor) of a 12-pair SiO_2_/TiO_2_ top DBR sample is evaluated using a high-power above-band excitation with a continuous-wave laser operating at 785 nm under cryogenic conditions (4 K). The mean Q-factor is plotted as a solid purple circle, with the error bar representing one standard deviation. Measurements are performed on four different devices with the same base widths. The peak *Q*-factor of ~15,000 is achieved at a base width of 3.9 μm

To achieve tunable quantum light sources, we employ a transfer-printing technique. Arrays of microcavity membranes with a size of 280 μm × 280 μm are fabricated by substrate removal and subsequently transferred onto a PMN-PT substrate using a rubber stamp (polydimethylsiloxane), as shown in Fig. 2a. Epoxy-based photoresist (SU-8) is used for the membrane bonding on the target substrate. A wide-field photoluminescence imaging technique is used to spatially overlap QDs to the cavities^34^. The positions of pre-selected single QDs are determined with respect to alignment marks using photoluminescence imaging. As depicted in the inset of Fig. 2b, the fluorescence image of the QDs reveals bright spots that are distinctly visible at the center of the fabricated microcavity.

We have developed a heat reflow process to prepare the parabolic lensed defect. The photoresist is exposed around the target QDs using electron beam lithography. Reflowing at 160 °C for 5 minutes transforms the photoresist from disks into truncated spheres, and this topography is transferred to the SiO_2_ layer after inductively coupled plasma etching. Precise control over the etching selectivity between the photoresist and SiO_2_ is achieved by employing different etching chemistries. This enables the fabrication of SiO_2_ defects with desired aspect ratio. Detailed fabrication processes are provided in Supplementary Information Section 2.

Figure 2c shows a cross-section scanning electron microscope (SEM) image of a typical microcavity that containing a parabolic lensed defect. Notably, in contrast to epitaxial GaAs/AlGaAs layers used as the top DBR in ref. ^32^, dielectric layers faithfully maintain the curved profile even after reaching a thickness of few micrometers. The high quality of the defect can be further evidenced by atomic force microscopy measurements in Fig. 2d, revealing a very smooth surface with a roughness of only ~1.3 nm.

To experimentally assess the influence of defect size on the optical quality factor, we fabricate defects with various base width for a systematic study. All the samples feature 12-pair layers in the top mirror and are excited with a 785 nm continue-wave laser. The measured Q-factor increases with base width (*B*) and reaches a plateau at *B* = 3.8 μm (Fig. 2e). A maximum Q-factor of ~15,000 was measured, and error bars represent the standard deviation among various cavities. It is worth noting that in an open cavity, a maximum Q-factor of 3.1 × 10^4^ (~6.6 × 10^6^) is reported without (with) passivation of the GaAs surface^35^. The observed gap between simulation and experiment in Q-factor is be mainly attributed to the presence of non-ideal dielectric layers produced by evaporation and the scattering/nonradiative recombination losses from GaAs surface. Further improvements can be achieved through the use of enhanced passivation methods, such as atomic layer deposition and the deposition of high-quality dielectric films.

### Strain tunability

**Fig. 3:**
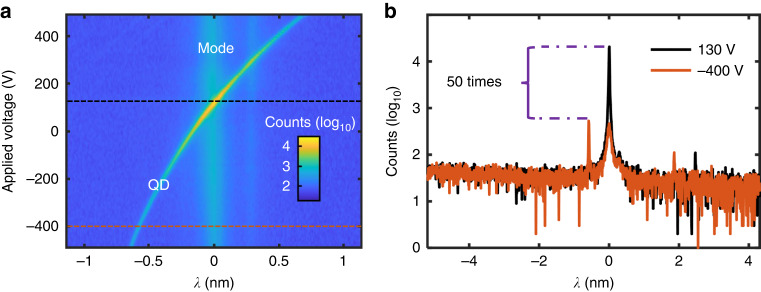
Wavelength tunability using strain. **a** Strain tuning plateau: The QD emission and cavity mode are clearly identified, scaled on a log10 axis. A wavelength shift of near 1.3 nm is achieved when sweeping the applied voltage from −500 V to 500 V. The thickness of PMN-PT (100) substrate used in this experiment is 250 μm. **b** Spectrum extracted from (**a**), corresponding to the dash black line (130 V) and solid brown line (−400 V). A near 50-fold enhancement is observed when tuned into resonance

Strain is a promising method to tune QD properties by integrating QDs onto piezoelectric actuators. However, this approach is challenging to implement in isolated structures such as micro-pillars^31^ or bullseye cavities^32^ due to the limited transfer of strain. The microcavity structures we have developed here are compatible with strain tuning. In the experiment, the strain tuning behavior is investigated by applying a voltage to the piezoelectric substrate while recording the QD emission. Figure 3a shows a photoluminescence (PL) mapping of the strain-induced shift for a single QD emission line in the vicinity of the cavity resonance. Both cavity modes and the emission peak are clearly identified, with their intensity plotted on a logarithmic scale for better viewing. Without tuning, the QD and the cavity are spectrally separated. When sweeping the voltage from −500 to 500 V, the emission peak also changes. Notably, when on resonance, a nearly 50× enhancement is observed, as shown in Fig. 3b. Concerning the QD’s emission, a tuning range of ~1.3 nm is achieved, which can be extended further by applying large voltages. The linewidth of the emission remains relatively constant when the QD emission far-detuned from cavity resonance. When a QD is coupled to the cavity, the lifetime ${{\rm{T}}}_{1}$ is reduced as a result of Purcell effect. This reduction in lifetime would, in principle, broaden the linewidth. However, this effect is beyond the resolution of our spectrometer. Notably, the cavity modes remain relatively constant throughout this process, which is a distinctive feature compared to temperature tuning. This stability can be attributed to the fact that the estimated biaxial strain-induced changes are insufficient to significantly alter the geometrical dimensions and dielectric constant of the cavity, and consequently, the cavity mode (see details in Supplementary Information Section 1-II). Due to the shield of metal electrodes, there is no electric field leaking. The spectral shift is solely the consequence of strain.

### Single photon emission in QD coupled to monolithic Fabry-Perot microcavity

To develop a highly bright, polarized single-photon source, we employ a charged exciton (CX) with circular polarization, and couple it to a geometrically birefringent monolithic Fabry-Perot microcavity in the Purcell regime. The observed mode splitting observed in the cavity is primarily induced by residual asymmetric uniaxial strain in the semiconductor materials and elliptical geometry introduced by fabrication, a phenomenon also reported in open cavities^36^. It worth noting that this splitting can be harnessed to overcome the 50% strain loss limit in resonant excitation when employing cross-polarization laser filtering schemes^17,22^.

**Fig. 4:**
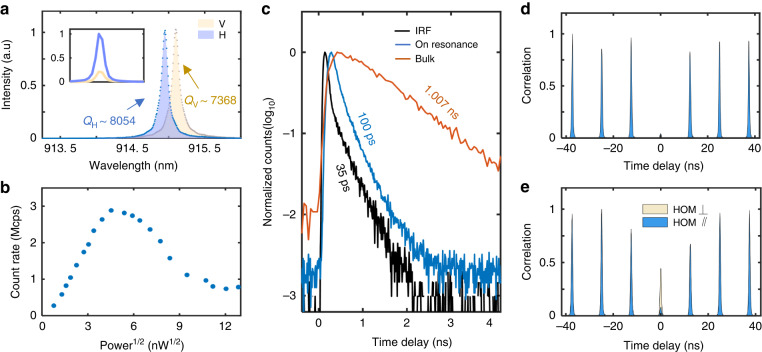
Characterization of a deterministically coupled single photon source. **a** Mode splitting and QD spectrum: Under high power excitation, two linear polarized fundamental modes, horizontally (H) and vertically (V) polarized, are identified using cross-polarized techniques. The inset shows that 0.85 of the photons are directed into the H mode, while the remain 0.15 are directed into the V mode when the QD emission (CX) is in resonance with the H mode. **b** The APD count rate of the single source is measured under pulsed resonant excitation with a repetition rate of 80.1 MHz. Rabi oscillation is observed, with the maximum population inversion reached at 25 nW, corresponding to a π-pulse. A count rate of ~2.88 Mcps (million counts per second) is recorded. **c** Time-resolved lifetime (*τ*) measurement: The CX lifetime histogram (blue line, *τ* ~ 100 ps) and the typical CX state lifetime curve in bulk material for reference (brown line, mean *τ* ~ 1.007 ns), in log10 scale, are depicted, thus *F*_p_ of ~9 is determined. Noted that seven dots in the same substrate. It is noteworthy that these measurements were conducted on seven dots within the same substrate. The instrument response function (IRF) is represented by the black line, with a *τ* ~ 35 ps. **d** Second order autocorrelation measurement of single photons from the deterministically-coupled QD. A value of *g*^(2)^(0) = 0.044(2) is extracted, where the uncertainty represents one standard deviation obtained from a double-Gaussian fit to the central peak area at zero delay. **e** HOM interference: The raw visibility of 0.811(3) is extracted from second-order correlation measurement of two consecutively emitted photons separated by ~12.48 ns

Figure 4a shows the PL of our microcavity modes with 7-pair top DBR under non-resonant excitation at high-power, which splits into two modes, horizontally- and vertically-polarized (H- and V-polarized), with a separation of ~55 GHz. The linewidths of the H and V modes are $\delta {\omega }_{H}$ = 40.7 GHz and $\delta {\omega }_{V}$ = 44.5 GHz, resulting in corresponding quality factors of 8054 and 7368, respectively. Theoretically, the spontaneous radiation rate of the exciton’s circularly polarized transition in this birefringent cavity is expected to be redistributed into *H* and *V* polarizations. When brought into resonance, the emission rate is faster than the off-resonance polarization with a factor of $1+4{(\Delta \omega /\delta \omega )}^{2}$^22^, where $\Delta \omega$ is the mode splitting and $\delta \omega$ is linewidth of the off-resonance mode. Hence, a high degree polarized spontaneous emission of ${\zeta }_{H}=\left[1+4{\left(\frac{\Delta \omega }{\delta {\omega }_{V}}\right)}^{2}\right]/[2+4{\left(\frac{\Delta \omega }{\delta {\omega }_{V}}\right)}^{2}]$=0.88 is predicted when we bring the QD into resonance with the cavity H mode. The *H* and *V* polarization components of the QD’s emission are shown in the inset of Fig. 4a.

To estimate the brightness, we conducted pulsed resonance fluorescence for CX under laser excitation at an 80.1 MHz repetition rate, assisted by a weak 785 nm continue-wave laser to achieve nearly blinking-free single photon emission. Figure 4b shows the detected single photon flux as a function of the driving laser power, revealing a complete Rabi oscillation curve. For this device, about 2.88 MHz is recorded by an avalanche photon detector, which we denote as the “π-pulse” condition. Considering the set-up efficiency (0.067), and avalanche photodiode correction factor (1.08) into account, an extraction efficiency of 0.58 is extracted. Detailed calibration of the system efficiency is described in Supplementary Information Section 3. Residual lasers are removed by a cross-polarization setup and a long-pass filter.

Furthermore, the radiative lifetime for the QD on resonance is shorten to ${\tau }_{{on}} \sim 100{ps}$ (blue line in Fig. 4c). Compared with the average lifetime for the QDs in the slab from the same area (red line in Fig. 4c), a Purcell factor of ~9 is achieved. As a result, the probability of emission into the H-polarized mode ${\beta }_{H}$, can be determined to be ${\beta }_{H}={{F}_{p}/(F}_{p}+1)\times {\zeta }_{H}=0.792$. The degree of photon polarization can be further boosted by increasing the cavity mode splitting, which can be achieved by using a defect microcavity with ellipticity (see Supplementary Information Section 1-III, Figure S2) or by incorporating thicker top DBR to increase the Q factor of the cavity.

To further assess the purity and indistinguishability of our single-photon source, the collected photons are directed to a fiber-based Hanbury Brown and Twiss setup. The second-order autocorrelation in Fig. 4d reveals *g*^(2)^(0) = 0.044(2) at zero-time delay, indicating clear photon antibunching and a high degree of single-photon purity. The non-vanishing peak at zero-time delay originates from a small amount of laser light leaking into the detection channels, as well as re-excitation events. Furthermore, the coherence of the single photons is measured using a Hong-Ou-Mandel (HOM) interferometer, with the time separation of 12.48 ns between the two emitted single photons, which is consistent with that of the laser pulses. Figure 4e shows the photon correlation histograms of normalized two-photon counts for orthogonal and parallel polarizations, indicating a raw HOM visibility of 0.811(3). Taking into account the imperfect single-photon purity and an unbalanced (52:48) beam-splitting ratio in the optical setup, we calculate a corrected photon indistinguishability of 0.922(4) (see Supplementary Information Section 3). This demonstrates that generated single photons based QD in microcavity are highly coherent.

## Discussion

In summary, we have developed a monolithic Fabry-Perot microcavity structure with the advantage of optimal exploitation of the Purcell effect, a compact footprint and integration capabilities. Given the rapid development of single photon sources with QD-in-microcavity technologies, it is useful to systematically compare the performance of our device with existing sources reported in the literature, as presented in Supplementary Information Section 4. In general, by deterministic embedding of a single QD into the microcavity, we have achieved high-performance single photon sources with simultaneous high extraction efficiency, high purity, and high indistinguishability. Moreover, the far-field characteristics of our microcavity exhibit Gaussian and convergent features, rendering our devices compatible and advantageous for integration with fiber networks. A feasible approach could involve directly coupling the device to a fiber^37,38^.

Considering future developments, firstly, our microcavity device, facilitated by its membrane structures, inherently lends itself to compatibility with electrical contacts. Charge stabilization or spin injection using electrical gated-devices can be directly implemented in our devices to realize low-noise single-photon emission^39^ or spin-photon entanglement/a linear cluster state. As discussed in ref. ^13^, it is anticipated that an entanglement length as long as 55 photons can be achieved by embedding the sources into microcavities with a feasible Purcell factor of 10. Secondly, strain tuning can also be employed to erase the spectral inhomogeneity between different QDs and address the fine structure splitting (FSS). These aspects are pivotal in the realization of high-performance source of entangled photon pairs. To achieve a cavity linewidth conducive for the extraction of both photons (biexciton and exciton), the device should be designed to have a Q factor in the range of 200–300, which yields a cavity bandwidth of ∼4 nm^35^. Thirdly, the operation wavelength for both QDs and photonic nanostructures can be translated to the telecom band by adjusting the capping layer of QDs and the thickness of the cavity and DBRs. This adaptability positions our devices for immediate applications in both fundamental physics and applied quantum technologies, such as quantum computing/communication with single-photon sources, generation of spin-photon entanglement, and the creation of linear cluster states for all photonic quantum repeaters^40,41^. Fourthly, by optimizing the DBR for higher reflectivity and minimizing GaAs surface scattering/nonradiative recombination losses using surface passivation, we can achieve higher Q factor and therefore strong coupling, which may open a route towards a photon–photon gate^42^. Most intriguingly, the simplicity and versatility of our cavity scheme open avenues for establishing a new manufacturing paradigm for quantum light sources, in which multiple types of solid quantum light sources (including semiconductor QDs, defects et al.) with different emitter materials and operating wavelengths could be co-manufactured on the same PMN-PT platform. This potential breakthrough could significantly advance scalable quantum photonic technologies in the future.

## Materials and methods

### Numerical simulation

The Q factor, mode volume, Purcell factor and mode profile were calculated by the Finite-Difference Time-Domain method. Perfectly matched layer domains are used to reduce the far-field reflection. The simulation time is set to be 10^6^ fs to ensure full convergence of the simulation. See detailed results in Supplementary Information Section 1-I and III.

### Sample growth

A semiconductor heterostructure was grown on a GaAs (100) substrate using a solid source molecular beam epitaxy (Veeco GENxplor system). The structure consisted of a 500-nm-thick sacrificial Al_0.8_Ga_0.2_As layer, 46-pair of GaAs/Al_0.95_Ga_0.05_As DBR, with low-density InAs/GaAs QDs embedded at the center of a 2-*λ* GaAs film.

### Supplementary information

Supplementary Material
