# Supplementary material for: Tunable quantum dots in monolithic Fabry-Perot microcavities for high-performance single-photon sources
Source: Light Sci Appl. 2024 Jan 30;13:33. doi: 10.1038/s41377-024-01384-7 (PMC10828388; doi:10.1038/s41377-024-01384-7)
Supplement: Supplementary file 1 — Supplementary Material [file 41377_2024_1384_MOESM1_ESM.pdf]

Supplementary Information For

Tunable quantum dots in monolithic Fabry-Perot microcavities for high-performance single-photon sources

Jiawei Yang[1#], Yan Chen[2,3,4#], Zixuan Rao[1], Ziyang Zheng[1], Changkun Song[1], Yujie Chen[1], Kaili Xiong[2,4], Pingxing Chen[2,4,5], Chaofan Zhang[3], Wei Wu[2,4,5], Ying Yu[1,5*], Siyuan Yu[1,5]

[1] *State Key Laboratory of Optoelectronic Materials and Technologies, School of Electronics and Information Technology, Sun Yat-Sen University, Guangzhou 510006, China*

[2] *Institute for Quantum Science and Technology, College of Science, National University of Defense Technology, Changsha 410073, China*

[3] *College of Advanced Interdisciplinary Studies, National University of Defense Technology, Changsha 410073, China*

[4] *Hunan Key Laboratory of Quantum Information Mechanism and Technology, National University of Defense Technology, Changsha 410073, Hunan, China*

[5] *Hefei National Laboratory, Hefei 230088, China*

**Corresponding author*: yuying26@mail.sysu.edu.cn

[#]These authors contributed equally to this work

Section 1 Theoretical analysis.

I. Geometry parameters sweep of Fabry-Perot microcavity.

To estimate the device's performance, we conducted a two-dimensional finite-difference time-domain (2D-FDTD) simulation. The main parameters considered were the base width (B), lensed-defect height (H), and SiO_2 spacer thickness (S), as shown in **Fig. S1a.** The device structure includes a 7-pairs top distributed Bragg reflector (DBR) and a 46-pairs bottom DBR, with stop band centers at a wavelength of λ_0=920 nm. The thickness of the GaAs spacer was set to $2\lambda_0/n_{GaAs}$, where n_{GaAs} represents the refractive index of GaAs.

The Fabry-Perot microcavity was designed as a miniaturized Fabry-Perot cavity, where the resonant wavelength exhibited a linear relationship with changes in the cavity length, with a slope of 1.7 nm/10 nm, as shown in **Fig. S1b**. Next, we investigated the influence of the defect height (H) on the device performance. It was found that as the height increased, the resonant wavelength decreased with a slope of -0.056 nm /10 nm. Although the Q-factor slightly degraded with increasing height, it still exceeded 15000 (**Fig. S1c**). Here, B and S were set as 4μm and 480nm, respectively. Regarding the base width (B), the simulation results revealed two distinct regimes at S=480nm and H=350nm, as shown in **Fig. S1d**. In the range of 2-3.5 μm, the Q-factor exhibited a logarithmic dependence on the base width. While in the range of 3.5-7 μm, it showed a close-to-linear dependence. As the base width expanded, the Q-factor increased and approached a value close to 16000 for larger B. However, an additional loss was observed around 2.7 μm, which aligned with experimental observations (**Fig. 2e** in the main text). All up, at S=480 nm, B=4

μm and H=350 nm, the quality factor (Q-factor) reached a maximum of 15614.

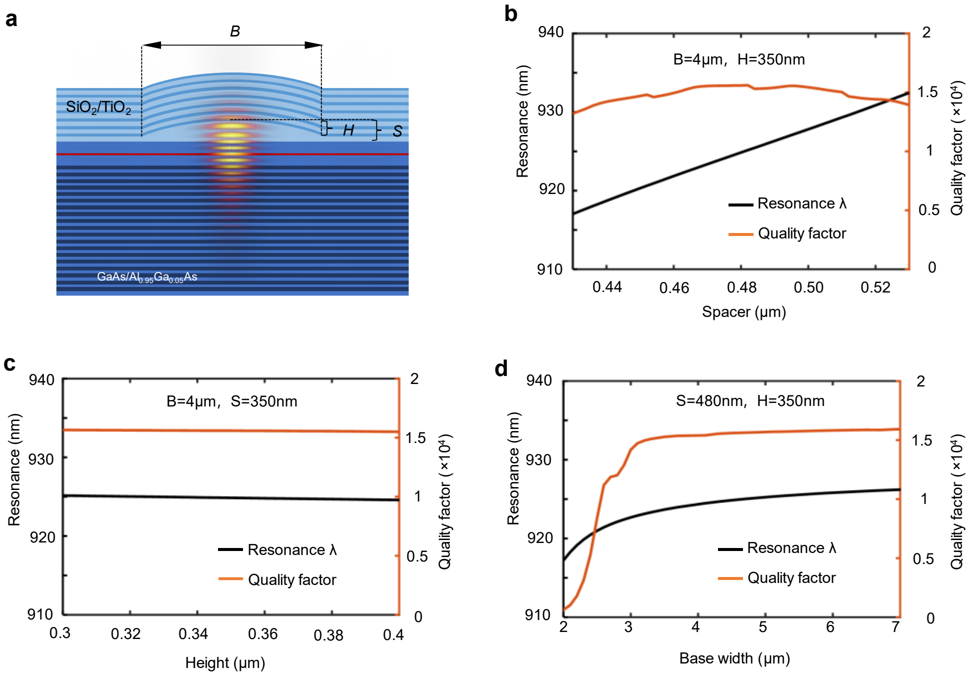

Figure S1. 2D-FDTD simulation and parameter sweep.

II. Strain engineering

We now consider an in-plane strain and show how they affect the exciton energy. Since the external strain is relatively small, the envelope functions of the electron and hole states change little as well as the electron-hole Coulomb interactions. The change of exciton energy ΔE_{ex} is therefore mainly determined by the single-particle energies $\Delta E_g = \Delta(E_c - E_v)$. The Bir–Pikus model[1] can be used to describe the effects of strain on valence states in zinc-blende structures. The Bir-Pikus Hamiltonian can be expanded in the six |j; jz⟩ states, specifically the heavy hole (HH) |3/2; ±3/2⟩, light hole (LH)|3/2; ±1/2⟩ and spin orbital (SO) |1/2; ±1/2⟩ states. This leads to the following 6 × 6 matrix.

$$HBP = \begin{pmatrix} P+Q & 0 & -\sqrt{2}S & R & S & \sqrt{2}R \\ 0 & P+Q & R^* & \sqrt{2}S^* & \sqrt{2}R^* & -S^* \\ -\sqrt{2}S^* & R & P-Q & 0 & \sqrt{2}Q & -\sqrt{3}S \\ R^* & \sqrt{2}S & 0 & P-Q & \sqrt{3}S^* & \sqrt{2}Q \\ S^* & \sqrt{2}R & \sqrt{2}Q & \sqrt{3}S & P & 0 \\ \sqrt{2}R^* & -S & -\sqrt{3}S^* & \sqrt{2}Q & 0 & P \end{pmatrix}$$

where

$$P = av(e_{xx} + e_{yy} + e_{zz})$$

$$Q = \frac{bv}{2}(e_{xx} + e_{yy} - 2\, e_{zz})$$

$$R = \frac{\sqrt{3}}{2}bv(e_{xx} - e_{yy}) - idve_{xy}$$

$$S = \frac{dv}{2}(e_{zx} - ie_{yz})$$

av, *bv*, and *dv* are the isotropic, biaxial, and shear deformation potentials respectively and e_{ij} are the strain components in the QDs. P characterizes the impact of isotropic hydrostatic strain and *Q* is associated with the biaxial strain. The influence of in-plane and off-plane strain anisotropy are accounted by *R* and *S*.

In self-assembled InAs/GaAs QDs grown on the (001) GaAs substrate, due to the significant lattice mismatch (7%) between InAs and GaAs, the biaxial strain is much greater than the shear strains. As a result, Q is significantly larger than |R| and |S|. Therefore, we can safely treat R and S as perturbations in the Hamiltonian. We calculate the first two (degenerate) hole states up to second order of R and S, and the single-particle energy of the two states is

$$Ev = Ev0 + P + Q + \frac{\Delta}{3} + \frac{2\Delta|S|^2 + (9Q+\Delta)|R|^2}{2Q\Delta} + \frac{3\sqrt{3}[S^2R^* + (S^*)^2R]}{2Q\Delta}$$

We neglect the valence-conduction bands (VB-CB) coupling for the simplicity and the energy of conduction band merely depends on hydrostatic strain:

$$Ec = Ec0 + ac(e_{xx} + e_{yy} + e_{zz})$$

Here we only consider the in-plane stress generated by the piezo substrate. The changes of *Pirkus* parameters under uniaxial stress along different directions are listed in Table S1. Using this table, by the *higher order* term, we can estimate the change of the band gap under stress as,

$$\Delta E_g = \Delta(E_c - E_v) = T_{-}\Delta p_{110}$$

Where T- is the slope of the bandgap for in plane strain and is expressed as

$$T_{-} = -(a_c - a_v)(S_{11} + 2S_{12}) + \frac{1}{2}b_v(S_{11} - S_{12})$$

It worth to note that the slope of the bandgap is not related to specific direction. In-plane strain [110], [100] or [010] applies the same rule.

Table **S1:** Change of strain tensor and Bir-Pikus model parameters (P , Q, R, S) under uniaxial stress (p) along different directions.

Stress direction	Strain	Parameters in Bir-Pikus model
[010]	$\Delta e_{xx} = \Delta e_{zz} = -S_{12}p$<br>$\Delta e_{yy} = -S_{11}p$<br>$\Delta e_{xy} = \Delta e_{yz} = \Delta e_{zx} = 0$	$P(p) = P(0) - av(S_{11} + 2S_{12})p$<br>$Q(p) = Q(0) - \frac{1}{2}bv(S_{11} - S_{12})p$<br>$S(p) = S(0)$<br>$R(p) = R(0) + \frac{\sqrt{3}}{2}bv(S_{11} - S_{12})p$
[100]	$\Delta e_{yy} = \Delta e_{zz} = -S_{12}p$<br>$\Delta e_{xx} = -S_{11}p$<br>$\Delta e_{xy} = \Delta e_{yz} = \Delta e_{zx} = 0$	$P(p) = P(0) - av(S_{11} + 2S_{12})p$<br>$Q(p) = Q(0) - \frac{1}{2}bv(S_{11} - S_{12})p$<br>$S(p) = S(0)$<br>$R(p) = R(0) - \frac{\sqrt{3}}{2}bv(S_{11} - S_{12})p$
[110]	$\Delta e_{xx} = \Delta e_{yy} = -0.5*(S_{11} + S_{12})p$<br>$\Delta e_{zz} = -S_{12}p$<br>$\Delta e_{xy} = -0.25*S_{44}p$<br>$\Delta e_{yz} = \Delta e_{zx} = 0$	$P(p) = P(0) - av(S_{11} + 2S_{12})p$<br>$Q(p) = Q(0) - \frac{1}{2}bv(S_{11} - S_{12})p$<br>$S(p) = S(0)$<br>$R(p) = R(0) + \frac{i}{4}dv\, S_{44}p$

III. Spontaneous emission rate redistribution.

The influences of mode splitting and linewidth of cavity on the spontaneous emission (SE) rate into two linear polarization direction, horizontally (H) and vertically (V) polarized, in a birefringent cavity are discussed in Ref. 1.

According to the reference, the ratio of photons funneled into the H polarization and V polarization is given by $1 + 4(\Delta\omega/\delta\omega_V)^2$: 1, where Δω represents the mode splitting and ω_V represents the linewidth of the V mode[2]. Hence, a degree polarized spontaneous emission of $\zeta_H = \left[1 + 4\left(\frac{\Delta\omega}{\delta\omega_V}\right)^2\right]/[2 + 4\left(\frac{\Delta\omega}{\delta\omega_V}\right)^2]$ is predicted when we bring QD into resonance with the cavity H mode. It can be observed that when the QD couples with H mode, the emitted single photons are predominantly H polarized. When the QD couples with V mode, the single photons are mostly V polarized, as shown in Figure S2a. The two polarization, H and V modes, are equivalent. Figure S2b shows the quality factor (Q) and mode splitting (Δλ) of cavities, while the difference between two modes is $\Delta Q = Q_H - Q_V$. Figure S2c and S2d demonstrate that a higher degree of SE rate into H polarization can be achieved when a circularly polarized transition coupled into H mode, as we enlarge the mode splitting and the Q-factor.

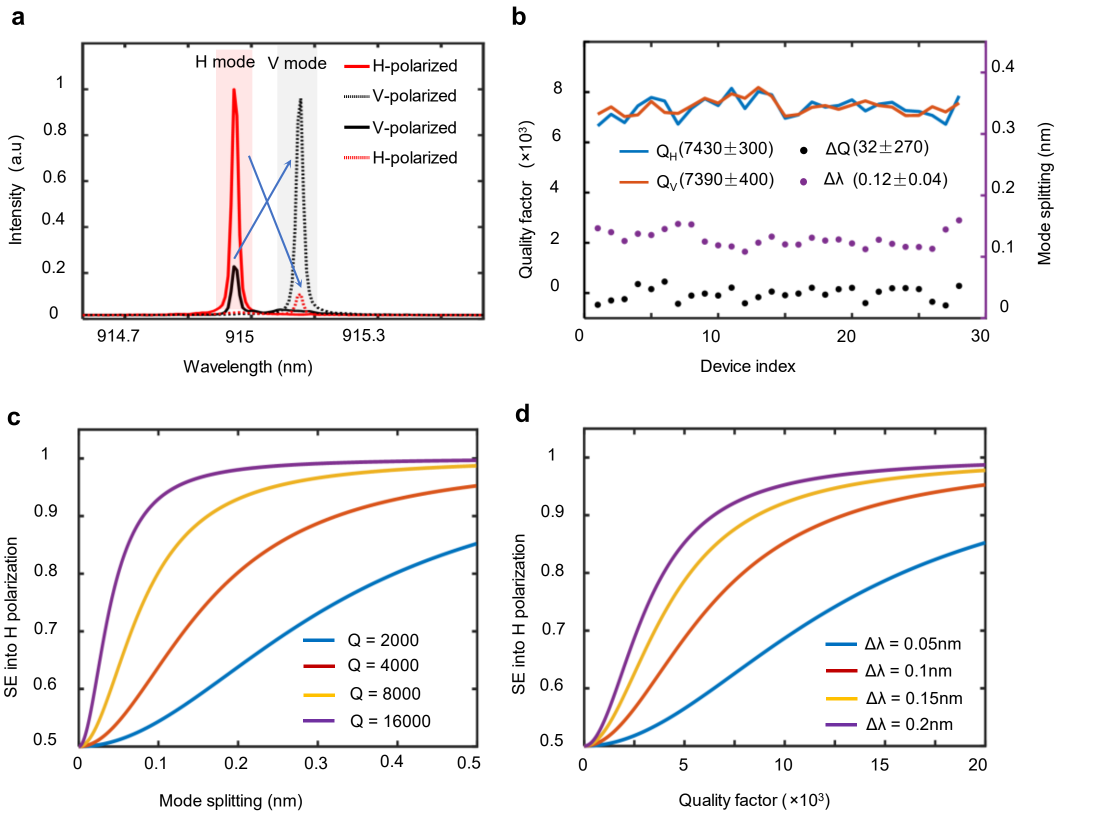

Figure S2. (a) The experimental polarization of the QD emission when coupled to H mode and V mode. The QD emission exhibits highly polarized and complies with the specific cavity mode. (b) The distribution of quality factor (Q) at different polarizations and the mode splitting (Δλ) of cavities. (c) Impact of mode splitting and quality factor on spontaneous emission.

Section 2 Device Fabrication.

I. Fabry-Perot microcavity fabrication.

A semiconductor heterostructure was grown on a GaAs (100) substrate using a solid source molecular beam epitaxy (Veeco GENxplor system). The structure consisted of a 500 nm-thick sacrificial $Al_{0.8}Ga_{0.2}As$ layer, 46 pairs of GaAs/$Al_{0.95}Ga_{0.05}As$ DBR, with low-density InAs/GaAs quantum dots (QDs) embedded at the center of a 2λ GaAs film (see **Figs. S3a, b**).

The fabrication flow is schematically shown in **Figs. S3c-i**. Firstly, an array of Ti/Au metal alignment marks is fabricated on the surface of the structure through a standard lift-off process (**Fig. S3c**). Secondly, a wide-field

positioning technique was employed to determine the QD position by pointing the maximum of the QD emission according to the two-dimensional alignment marks (**Fig. S3d**, see details in **Section 3**). Thirdly, to fabricate the lensed-defect, a 25 nm-thick SiN_x layer and 450nm SiO_2 layer were deposited at 100℃ using plasma-enhanced chemical vapor deposition (**Fig. S3e**). Then the photoresist (MaN-2400) was spin-coated and selectively exposed over the QDs using electron beam lithography (EBL) overlay (**Fig. S3f**). Heat reflow was performed by placing the sample on a hotplate at 160℃ for 5 minutes, causing the photoresist to liquefy and reshape into a truncated sphere. Subsequently, the substrate was transferred to a room-temperature hotplate, facilitating the rapid curing of the photoresist and forming a solid truncated sphere (Fig. S3g). For enhanced etch resistance and to achieve a higher SiO_2 defect, the sample can be further baked at 100℃ for an extended duration. The paraboloid lensed-shaped defect was then defined using inductively coupled plasma etching (ICP-RIE) with a CF_4-based etchant, with an etching rate of approximately 60 nm per minute (**Fig. S3h**). Finally, a SiO_2/TiO_2 top DBR was evaporated by electron beam evaporation to complete the Fabry-Perot microcavity (**Fig. S3i**).

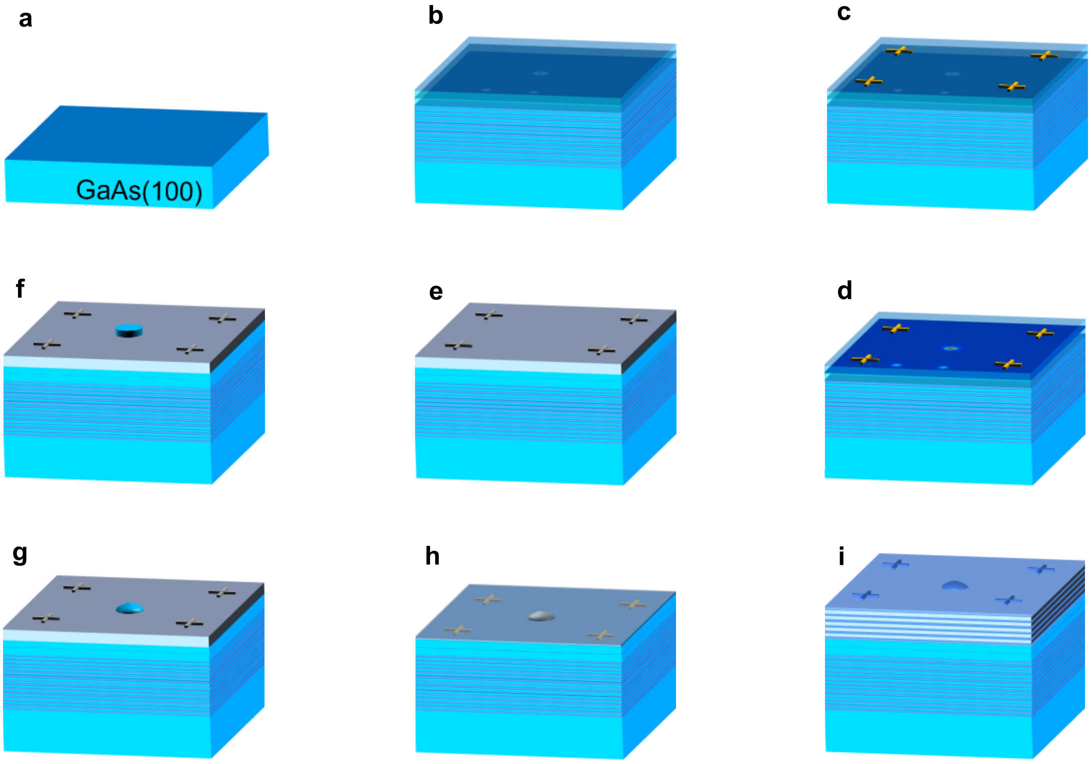

Figure S3. Cavity fabrication flow

II. Integration with piezoelectric actuator.

To integrate the Fabry-Perot microcavity with a piezoelectric actuator, we extracted a film with deterministically coupled QD-in-microcavity devices from the fabricated chip and bonded it to a 250 μm-thick PMN-PT(100) substrate. In details, firstly, we spin-coated AZ-2070 on the sample and exposed 280 μm × 280 μm mask using optical direct-write lithography (**Fig. S4b**). ICP-RIE was then employed to etch the SiO_2/TiO_2 distributed Bragg reflector (DBR) and the GaAs/AlGaAs DBR using CF_4 and $SiCl_4$-based etchants, respectively (**Fig. S4c**). Secondly, we filled the gaps between the films with paraffin and bonded them to a silicon substrate. The GaAs substrate, with a thickness of approximately 300 μm, was subtracted using physical milling. The remaining GaAs layer (~50 μm) was removed using a citric acid-based solution in a water bath at 27 ℃ for approximately 2 hours. The etching process was blocked by the AlGaAs layer (**Fig. S4d**). The chemical etching solution was prepared as follows: (a) dissolving citric acid monohydrate in deionized water with a mass ratio of 1:1, and (b) mixing the 1:1 solution with

30% H_2O_2 in a volume ratio of 3:1. The processed sample was immersed in acetone to dissolve the paraffin and separate the flakes. Subsequently, a single flake was picked up using PDMS and pressed onto a PMN-PT(100) substrate with SU-8, followed by baking for 10 minutes (**Fig. S4e**). Finally, the film with the Fabry-Perot microcavity was integrated with a piezoelectric actuator (**Fig. S4f**).

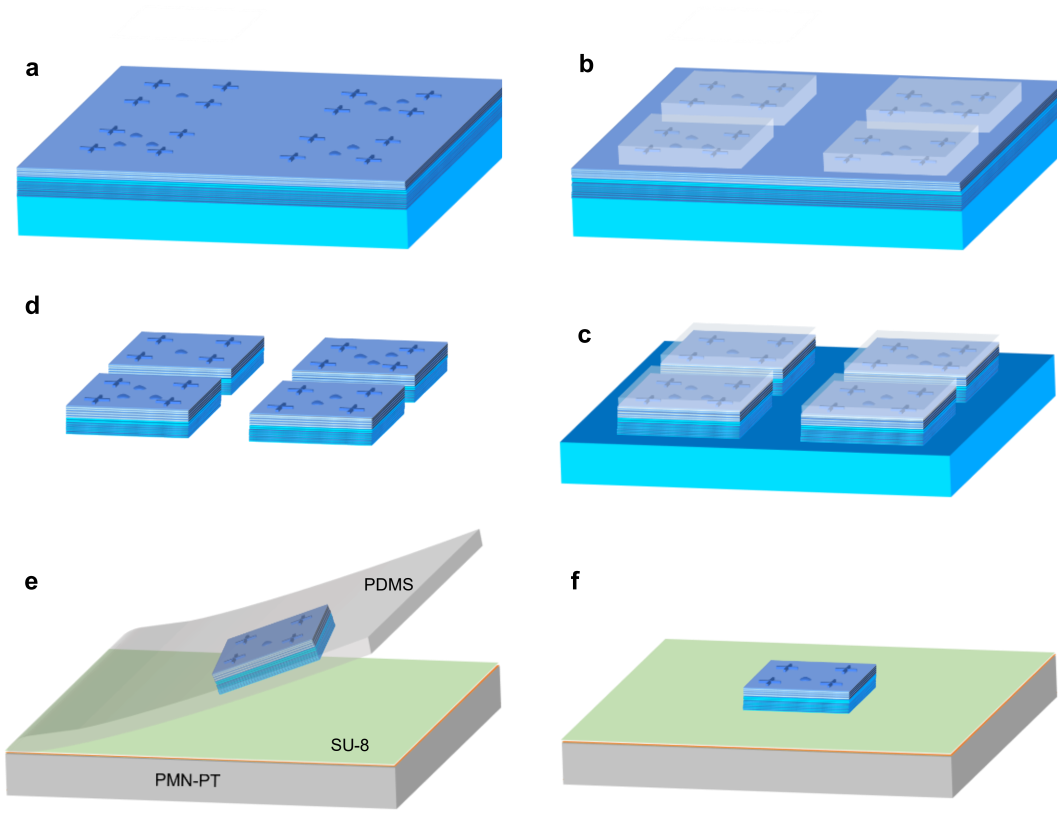

Figure S4. Integration to piezoelectric actuator

Section 3 Optical setup and source performance estimation.

I. Wide-field photoluminescence positioning setup.

Nano-positioning techniques are widely employed to deterministically fabricate a single QD into specific photonic structures. In this study, we utilized a wide-field photoluminescence (PL) positioning technique (see **Fig. S5**). The positioning setup consists of two main parts: excitation and imaging. The procedure is as follows:

(a) The QDs are excited using a high-power blue LED with a wavelength of 445 nm. The emission light is reflected by a dichroic beam splitter, which allows wavelengths longer than 650 nm to pass, extra filters such as notch filters centers at 785 nm, 830 nm and 880 nm are inserted before the camera. This ensures the PL signals captured by an electron-multiplying charge-coupled device (EMCCD) camera are free from residual blue light and background signals, such as PL from GaAs and the wetting layer. The resulting image obtained by the camera reveals bright spots that correspond to the positions of the QDs.

(b) The blue LED is turned off, and a white LED is switched on to illuminate the alignment marker. An image is captured to identify the position of the marker.

(c) The sample is then moved to the next region using a nano-positioner, and steps (a) and (b) are repeated to acquire data from additional regions of interest.

The two sets of images are processed separately to extract position information. By calculating the relative position between each QD and the corresponding alignment marker, the absolute position of each QD can be determined. Moreover, for improved spectral alignment, a single QD can be selectively excited, and its emitted spectrum can be recorded by directing the PL signal to a spectrometer.

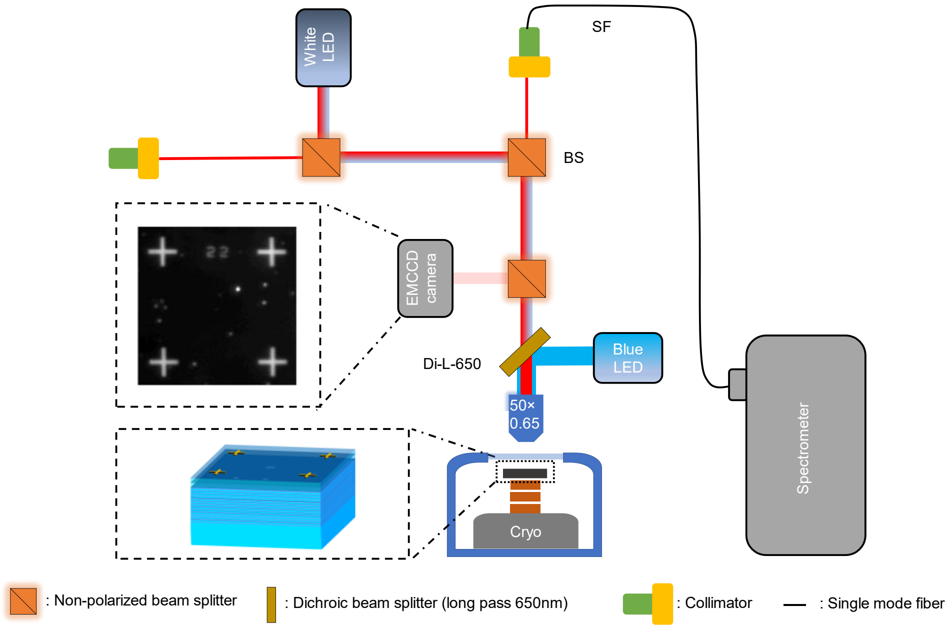

Figure S5. Wide-field photoluminescence positioning

II. Resonant excitation and performance estimation.

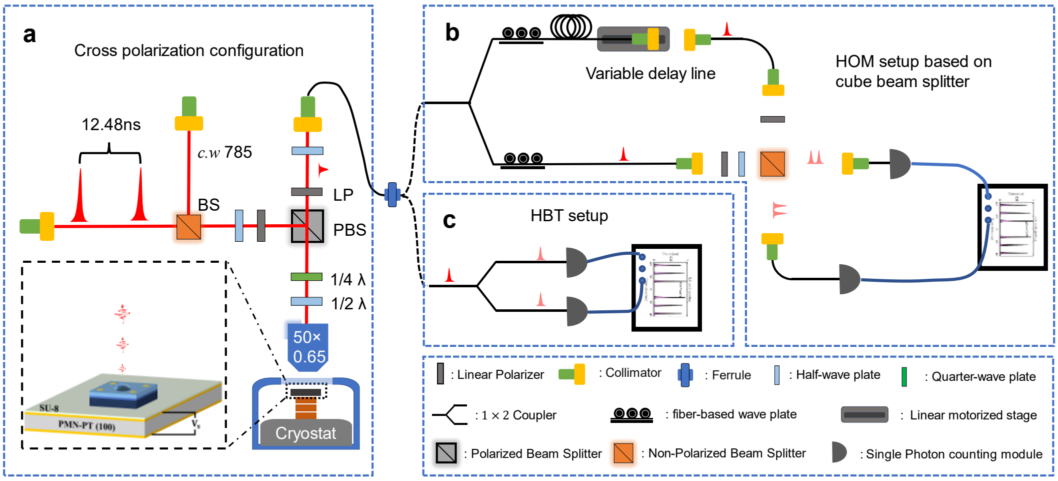

Figure S6. Resonant excitation and quantum optics experiment setup

Resonant Excitation Setup: The setup for resonant excitation is displayed in **Fig. S6a**. Resonant laser pulses with a separation of 12.48 ns are reflected by a polarizing beam splitter to excite the QD. The polarization axis is adjusted

by a half-wave plate to match the V-polarized mode. The optimization of excitation laser suppression can be achieved by making slight modifications to an additional quarter-wave plate. Additionally, to stabilize the QD environment and minimize blinking, a weak continuous-wave laser at 785 nm with a power level of 3 nW is used for assisting excitation.

Hong-Ou-Mandel (HOM) Setup: The photons collected by a single-mode fiber are directed to a HOM setup based on a cube beam splitter (R:T=52:48), enabling two-photon interference (**Fig. S6b**). A fiber-based wave plate is utilized to maximize and balance the photon flux in the two input arms. Synchronization of the successively emitted photons is achieved by accurately adjusting a variable delay line with a linear motorized stage. A half-wave plate is also inserted to rotate the polarization of one channel, allowing the observation of distinguishable and indistinguishable interference.

Single-Photon Purity Measurement: To assess single-photon purity, the second-order autocorrelation is measured by connecting the output fiber to a fiber-based Hanbury Brown and Twiss (HBT) setup (**Fig. S6c**).

Extraction efficiency: The transmittance of the optical setup is calibrated by coupling a resonant linear-polarized continuous-wave laser into the system via the ferrule in figure S6, and its detection is performed at the front of the objective. The half-wave plate is adjusted to maximize the transmittance, resulting in a transmittance value of $\eta_o \sim 0.37$. The transmittance of the optical window, η_w, is determined to be approximately 0.92. Consequently, the overall optical transmittance is calculated as $\eta = \eta_o \times \eta_w = 0.3404$. Considering a detection efficiency of about 0.3, a coupling efficiency of approximately 0.66 to the single-mode fiber, and a correction factor of 1.08 for high count rate detection with an avalanche photodiode (APD), the estimated extraction efficiency, η_e, is approximately 0.58.

Corrected photon indistinguishability: Two photon interference is determined as $1\text{-}A_{\parallel}/A_{\perp}$,where $A_{\parallel}$ and $A_{\perp}$ are the area of central peak under perpendicular and parallel polarized two photon interference fringes. Non-vanishing $g^2(0)$, non-perfect classical interference visibility ($1\text{-}\varepsilon$) and unbalanced beam splitter (R:T=52:48) leads to deterioration of two photon interference visibility in our experiment. Central peak area[3]

$$A = N\eta\{(R^3T + T^3R)[1 + 2g^2(0)] - 2(1-\varepsilon)^2R^2T^2V\}$$

N is the photon generation rate, η is the combined two photon generation and detection efficiency, V is the intrinsic interference visibility, V=0 for distinguished photons and V=1 for ideal indistinguishability. Thus, the corrected visibility can be derived as

$$V_c = \frac{1}{(1-\varepsilon)^2}\frac{R^2+T^2}{2RT}(1 + 2g^2(0))V_{raw}.$$

For our setup, $1\text{-}\varepsilon \sim 0.98$, substituting all the parameter in above formula, a corrected visibility of 0.922 is extracted.

Section 4 Comparison of device performance

I. State of the art for high-performance single photon sources.

Table S2: The state of the art for high-performance single photon sources.

Photonic structures	Extraction efficiency	Indistingui-shability	Tunability	Device yield	Purcell factor
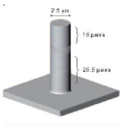 micro-pillar[4]	66%	98.5%	no	low	6.3
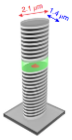 polarized micro-pillar[2]	60%	97.5%	no	low	17.8
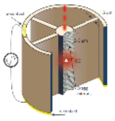 cross micro-pillar[5]	65%	99.5%	no	medium	7.6
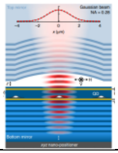 open cavity[6,7]	53%/ 71.7% (end to end)	96%/ 98.6%	yes	medium	10/ < 18
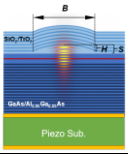 monolithic F-P cavity integrated with piezo, This work	58%	92%	yes	high	9

II. Strain tuned quantum dot

We have summarized the performance of commonly used strain-tuned quantum dot devices, displayed in Table S3. The strain-tuning technique can be applied to quantum dots at different wavelengths, with the largest tuning range demonstrated in Ref. 9, a quantum dot embedded in thin films, reaching up to 7 nm. For nanowires, in Ref. 11, the tuning range increases from 0.39 nm to 1.6 nm after depositing a 20 nm-thick SiN_x and a 200 nm-thick SiO_2 using plasma-enhanced chemical vapor. By filling the area around the pillar with SU-8, the authors of Ref.12 achieved a tuning range of 0.49 nm in shallow-etched micro-pillars with a Purcell factor of 4.4. These further illustrates that the thin-film structure is more conducive to strain transfer. Tuning range of 0.53 nm was also achieved in a thin hybrid circular brag grating (CBG), 125 nm. However, the indistinguishability was only 0.22 and time-post-selected indistinguishability reached 0.65. In such a thin film, quantum dots are sensitive to the defect in surface and the defect produced by plasma etching also had great impact. In contrast to resonant excitation, the use of two photon excitation in Ref. 14 and 15, where the excitation wavelength is different from the signal, allows for effective filtering of background laser using notch filters, resulting in a clean single photon emission.

Table S3: Strain tuned quantum dot devices

cavity	method	λ(nm)	t(um)	Δλ(nm)	$g^2(0)$	HOM	η	Fp
Photonic crystal[8]	Uniaxial	915	0.53	0.45	-	-	-	-
Membrane[9]	Mechanical	895	< 1	7	-	-	-	-
Membrane[10]	Biaxial	1548	6.43	0.28	-	-	-	-
Nanowire[11]	Biaxial	885	-	0.39/1.6	-	-	-	-
Micro pillar[12]	Biaxial	908	30	0.49	0.07	-	-	4.4
Cavity-Waveguide[13]	Biaxial	873	30	0.85	-	-	-	-
Hybrid CBG[14]	Uniaxial	916	0.125	0.53	0.0015	0.22	0.065	2.5
Parabolic cavity[15]	Biaxial	795	0.62	0.4	0.013	-	-	-
This work	Biaxial	915	9	1.3	0.043	0.922	0.58	9

Reference

1. Bir, G. L. & Pikus, G. E. Symmetry and strain-induced effects in semiconductors. (1974).
2. Wang, H. et al. Towards optimal single-photon sources from polarized microcavities. *Nature Photonics* **13**, 770–775 (2019).
3. Santori, C. et al. Indistinguishable Photons from a Single-Photon Device. *Nature* **419**, 594–597 (2002).
4. Ding, X. et al. On-Demand Single Photons with High Extraction Efficiency and Near-Unity Indistinguishability from a Resonantly Driven Quantum Dot in a Micropillar. *Physical Review Letters* **116**, 020401(2016).
5. Somaschi, N. et al. Near-optimal single-photon sources in the solid state. *Nature Photonics* **10**, 340–345 (2016).
6. Tomm, N. et al. A bright and fast source of coherent single photons. *Nature Nanotechnology* **16**, 399–403 (2021).
7. Ding, X. et al. High-efficiency single-photon source above the loss-tolerant threshold for efficient linear optical quantum computing. ArXiv:2311.08347 (2023).
8. Sun, S. et al. Strain tuning of a quantum dot strongly coupled to a photonic crystal cavity. *Appllied Physics Letters* **103**, (2013).
9. Chen, Y. et al. Highly-efficient extraction of entangled photons from quantum dots using a broadband optical antenna. *Nature Communication* **9**, 1–7 (2018).
10. Zeuner, K. D. *et al.* A stable wavelength-tunable triggered source of single photons and cascaded photon pairs at the telecom C-band. *Appllied Physics Letters* **112**, (2018).
11. Elshaari, A. W. et al. Strain-Tunable Quantum Integrated Photonics. *Nano Letters* **18**, 7969–7976 (2018).
12. Moczała-Dusanowska, M. et al. Strain-Tunable Single-Photon Source Based on a Quantum Dot-Micropillar System. *ACS Photonics* **6**, 2025–2031 (2019).
13. Hepp, S. et al. Purcell-enhanced single-photon emission from a strain-tunable quantum dot in a cavity-waveguide device. *Appllied Physics Letters* **117**, (2020).
14. Moczała-Dusanowska, M. et al. Strain-Tunable Single-Photon Source Based on a Circular Bragg Grating Cavity with Embedded Quantum Dots. *ACS Photonics* **7**, 3474–3480 (2020).
15. Lettner, T. *et al.* GaAs Quantum Dot in a Parabolic Microcavity Tuned to 87Rb D1. *ACS Photonics* **7**, 29–35 (2020).
